# Forum: COVID-19 Dispatches

**DOI:** 10.1177/1532708620953190

**Published:** 2021-02

**Authors:** Li Lu, Srinivas Lankala, Yuan Gong, Xuefeng Feng, Briankle G. Chang

**Affiliations:** 1 Beijing Normal University, China; 2 English and Foreign Languages University, Hyderabad, India; 3 Massey University, Auckland, New Zealand; 4 Hangzhou Normal University, China; 5 University of Massachusetts Amherst, USA

**Keywords:** COVID-19, viruses, pandemic, global crisis

## Abstract

COVID-19 pandemic is the first truly global crisis in the digital age. With death count worldwide reaching 586,000 merely 7 months after its first outbreak in China in late December 2019 and 13.6 million cases reported in 188 countries and territories as of July 2020, this ongoing pandemic has spread far beyond domain of world health problem to become an unprecedented challenge facing humanity at every level. In addition to causing social and economic disruptions on a scale unseen before, it has turned the world into a site of biopolitical agon where science and reason are forced to betray their impotence against cultish thinking in the planetary endgame depicted in so many dystopian science fictions. It is in this context that this forum offers a set of modest reflections on the current impacts incurred by the COVID-19 virus. Blending ethnographic observations with theory-driven reflections, the five authors address issues made manifest by the crisis across different regions, while keeping their sight on the sociopolitical problems plaguing our life both individually and collectively. Taken together, they provide a grounded documentary for the archive that the COVID-19 virus is making us to construct.

## Coronavirus as a Ghost Writer of *Envoi* (Li Lu)

The apparition of these faces in the crowd.

—Ezra Pound, “In a Station of the Metro”

The French word *envoi* is polysemic, defined as dispatch, the action of sending, something that is sent, a poetic dedication or dedication of a literary work, and the marking of the beginning of a process. This article is a dispatch from Hubei, China, based on the author’s 4-month stay in his hometown Qianjiang, a small city in the middle of Hubei, during the Coronavirus pandemic. Firsthand observations sent from the epicenter give us a clear picture of what the coronavirus has done. Moreover, this article argues that the coronavirus marks a spectral moment in which a repressed trauma returns. There have been fierce debates on the origins of the coronavirus and the political, economic, and social significances of the pandemic. Popular representations of the coronavirus which isolate, stigmatize, and terrify the Other are symptoms of a returning trauma, which is caused by bodily memories of being victims in past disasters. A Derridean reading of *the envoi* highlights the inherent failure of sending: What is sent can always be held up by a malfunctioning in the process of the sending or postal system, and the meaning of the trauma is lost. This traumatic failure results in a repetition in representation and the return of what is sent to the writer/sender. Proposing a supplement, this article foregrounds bodily knowledge acquired through social and political trauma by virtue of fear of the coronavirus. This fear of what is familiar reminds us of the feeling of the uncanny. According to Freud and Derrida, the uncanny is related to the spectral working of a hidden desire that repeatedly returns as a haunting body, representation, and history. This line of thinking helps us to better understand conflicting representations of the coronavirus.

The coronavirus is a ghost. This is not merely a metaphorical proposition; this is accurate in the sense that the coronavirus instantiates our phantasms, fears, and desires toward ghosts. In this regard, Derrida’s *Specters of Marx* provides us with a basic framework for understanding the coronavirus as a ghost. The first teaching of Derrida is that ghosts do not come at just any time but in spectral moments that do not belong to time. By pointing precisely to the present or now-time, Derrida (2006) regards spectral time as “a disjointed now that always risks maintaining nothing together in the assured conjunction of some context whose border would still be determinable” (p. 1). Second, a ghost is a phenomenon in the game of repetition and difference. Neither exclusively situated in life nor in death, neither visible nor invisible, a ghost is “the frequency of a certain visibility. But the visibility of the invisible. And visibility, by its essence, is not seen, which is why it remains *epekeina tes ousias*, beyond the phenomenon or beyond being” (Derrida, 2006, p. 125). Third, to “make oneself fear” is essentially ineluctable in the experience of a ghost. One becomes frightened of a ghost “on the condition that one can never distinguish between the future-to-come and the coming-back of a specter” (Derrida, 2006, p. 46). In other words, what one fears is not the ghost, but the fear, imagination, and one’s subject inspired by the ghost. Finally, “a ghost never dies, it remains always to come and to come-back” (Derrida, 2006, p. 123). Whatever repression the dead may suffer, the return of the dead is anticipated, and “this being-with specters would also be, not only but also, a politics of memory, of inheritance, and of generations” (Derrida, 2006, p. xviii). In light of Derrida’s framework on specters, once the coronavirus finds a host, it starts to live a ghostly life.

The coronavirus pandemic irrupted during a time of turmoil. As the Chinese president Xi Jinping has expressed, the world is experiencing profound shifts unseen in a century. While the trend of globalization is markedly receding, nationalism, popularism, and isolationism are on the rise. The eulogic discussions of “Chimerica,” a popular term coined by the British epidemiologist Neil Ferguson in 2007, are being replaced by the theories and practices of the China–U.S. decoupling. The trade dispute between China and the United States puts an end to the Chinese ideal of Great Harmony in the world. In addition, as his campaign slogan “America First” shows, Donald Trump epitomizes the idea of American exceptionalism. In traditional Chinese thinking, famine, natural disasters, and plague happen when the political order or legitimacy are out of joint. During this disjointed moment, a plague was anticipated, even fabricated before it came. According to a widely circulated story in the We-media during the height of the coronavirus, Wang Yongyan, an academician specializing in Chinese medicine at the Chinese Academy of Engineering, predicted half a year ago that a plague would come after the *dongzhi* (Winter Solstice), one of the 24 Chinese solar periods. In addition, he predicted that the plague would last until next spring. In hindsight, rumors about a new virus were spreading right after the *dongzhi*. Or, simply put, divination went hand in hand with the plague during a time of disjointing, disjunction, or disproportion.

While scientists are still trying to track down patient zero, conspiracy theories about the origins of coronavirus have been spreading. Bat soup and biological warfare are on the top of the list of suspected criminals. Like ghosts, the coronavirus takes shape in the game of visibility and invisibility. In this game, ways of seeing determine how a virus can be understood. Approximately made up of 0.125 microns, the coronavirus can only be seen under an electron microscope, made visible with the help of scientific equipment and representations. In contrast, a poet like Ezra Pound sees the invisible through his gifted imagination. His imaginative inspiration and aesthetic reflection allow his keen observation to become a line of poetic beauty and philosophical complexity. Through this form of observation, an invisible apparition becomes visible in the faces of the crowd. Similarly, the depiction of a fictional killer-virus called Wuhan-400 in Dean Koontz’s 1981 novel *The Eyes of Darkness*, re-gained popularity among those who regard it as an imaginative depiction of the coronavirus. In line with this imaginative depiction, Mr. Wang, well-trained in traditional Chinese medicine, claimed that his prediction was based on his reading of the *xiang* (image) of the sky, earth, plants, animals, and human beings. Visible to the naked eye, *xiang* functions as the visible traces from which an invisible plague becomes visible to an expert in traditional Chinese medicine. In other words, with scientific support, talent, and training, people are able “to see this invisibility, to see without seeing, thus to think the body without body of this invisible visibility” (Derrida, 2006, p. 187). In this way, the ghostly nature of the coronavirus lies in the different frequencies of its visibility.

However, despite our faith in being able to depict, and make distinctions between, the invisible and the visible, the way of seeing the coronavirus, especially in this time of turmoil, is politically conditioned and manipulated. When I took a night bullet train to Wuhan with my family for vacation, it felt like an ordinary Chinese family reunion trip during the Spring Festival: carriages packed with passengers, luggage, excitement, anxiety, and weariness in the air. One of the reasons for the peaceful atmosphere was that China and the United States had signed a trade agreement a few days before, sending a false message to the world that rationality and peace would return. One thing was markedly noticeable on the train: Most passengers wore a facial mask for fear of an officially unidentified but unofficially SARS-like virus. To my surprise, a line of masked faces was greeted at the exit by the smiling faces of relatives or friends, the indifferent faces of railroad workers, and the shrewd faces of barkers at the Hankou Railway Station. This lack of consistency indicates that aspects of the coronavirus were kept secret. Furthermore, this scenario at the station reminded me of a horrifying scene in the film *The Cassandra Crossing*, an eye-opening disaster thriller for my generation directed by George P. Cosmatos. In this harrowing film, an international express carrying a virus-infected terrorist approaches a station at night. When the train reached the station, the passengers, who were kept from the truth, were confronted with members of the U.S. army in white biological hazard protective suits lined up on both sides of the platform. In both cases, the dynamic of the visibility and the invisibility of a virus was of political significance. The facial masks and the protective suits were used not only to protect people from a virus but also to make the secret of the virus both visible and invisible. In other words, political manipulation complicates the ways that a virus is seen and how the coronavirus, in particular, is seen as a political ghost.

The coronavirus pandemic frightened people because it looked like the return of a specter, namely SARS. Because of its fatality and residua, SARS remains an unresolved trauma for many Chinese. At the early stages of the coronavirus pandemic, what was most frightening was its assumed high fatality rate. Similarly, the short notice given for the lockdown of Wuhan, a huge city of more than 10 million residents, sent a clear message to everyone that the novel coronavirus was the Grim Reaper. Corona, the brand of the first car I owned and of the beer I had on my first visit to a Mexican restaurant, was colored by images of a fearful virus, deserted streets, calm officials on TV news channels, and panicking crowds in Wuhan hospitals caught on video by the We-media. Unlike the countries who proposed or actually enacted herd immunity, the Chinese authorities imposed very tough immunity measures, a lesson learned from the 2003 SARS pandemic, when highways, the railway station, and docks in my hometown were closed overnight. Nursing homes were under quarantine; no visitors were allowed in. Local authorities advised avoiding public gatherings, including public square dancing and playing *majiang*. The most popular forms of social activity, especially for retired people, were no longer available.

After the initial panic, it was discovered that the fatality rate of the coronavirus was much lower than SARS. According to the World Health Organization, the SARS mortality rate worldwide was about 11%. In early February, the Chinese authorities claimed that the coronavirus mortality rate in Wuhan was about 5%. Subsequently, what elicited fear in the population was the future-to-come, particularly in the form of social unrest. On one hand, stricter quarantine measures were implemented: All roads were quickly blocked with cranes or tankers or stones; vehicles’ use was not allowed, unless a special permit was issued; all grocery shops, markets, restaurants, and hotels were shut down; residents were not allowed to exit their residential areas except for grocery shopping at an arranged supermarket. In addition, central and local authorities watched closely for other concerns, such as food shortages and the inflation of prices. Thanks partly to its rich agricultural products in a land of fish and rice, the impact of the coronavirus on food supply and prices did not affect my hometown. However, under the restrictions put in place, my hometown looked like a ghost town, and the uncertainty of the future frightened people of all social strata.

In fact, what people fear most is that the coronavirus will never die and will come back again and again, either in the form of a future-to-come or a return of the dead. Regardless, despite the medical or political ambition to eradicate the coronavirus, we might have to accept the fact that the virus will co-exist with us forever. For instance, the coronavirus has been mutating, and the way the coronavirus replicates itself in the cells of other organisms is ubiquitous. This mechanism of repetition and difference functions both literally and metaphorically. On one hand, the coronavirus reproduces itself through difference. Merely a collection of genetic materials that seems to think with/like a human once it infects its host, the virus induces a feeling of the uncanny, a topic to which I will return later. In addition, news sources reported that infected patients tested positive again after they had been released from the hospital. Robert Redfield, Director of the U.S. Centers for Disease Control and Prevention, admitted that some deaths from coronavirus have been discovered posthumously (CNN, 2020). In other cases, the coronavirus acted like a whimsical tyrant who inadvertently signed a death sentence. For example, the only cases of death in my residential area was an old couple who lived in an apartment very close to that of my parents. They got infected by their son and daughter who came back from Wuhan. What remained a mystery was that the son and the daughter had stayed with their parents for more than 20 days, much longer than the latent period of coronavirus. Days after they were hospitalized, they died one after another.

On the other hand, the coronavirus reproduces difference in its host organisms. The neighborhood my mother lives in is an acquittance community and an aging society. Cadres and volunteers from the neighborhood committee have diligently attended to the needs of the old. Aware of the higher fatality rate of the old, an ageist exhortation to quarantine was broadcast repeatedly through a portable loudspeaker placed at the gate of the neighborhood committee building. As stigmatized targets, senior residents were susceptible to the emotion of shame and, for this reason, chose to stay at home. The use of broadcasts and the instigation of shame illustrates how the coronavirus (re)produces, moderates, and polices the line between the public and private spheres.

The coronavirus also changed the affective, moral, and power economy of the family. The Spring Festival is supposed to be the perfect time for a temporary family reunion of joy and harmony. When the lockdown continued longer than everyone expected, generational conflicts broke out. In extreme cases, the political infected families while they were trying to contend with the coronavirus during quarantine. For example, Fang Fang, a veteran Chinese writer who lived in Wuhan, posted her thoughts on life in quarantine on her or her friend’s Weibo account. Those posts were later collected and published under the title *Wuhan Diaries*. Public opinion on those posts varied and eventually led to a political debate between left-wing and right-wing netizens, eventually affecting family members who conflicted in their attitudes toward the *Wuhan Diaries*.

Along with the coronavirus, the memory of personal, generational, and political traumas returned. SARS, the Cultural revolution, natural disasters, and national humilities were recurring themes in representations of the coronavirus. The suffering and trauma in the epicenter deserve an envoi/dedication, and efforts have been made to achieve this goal, such as daily national and international coverage, Fang Fang’s *Wuhan Diaries*, and We-media postings. In these kinds of representation, a rhetoric of “suffering as sublime” is usually at play. In addition, stigmatizing the suffering of Others, or blaming the Other for one’s suffering, is another kind of dedication. Both kinds of representations of the coronavirus attempt to take the moral higher ground by attempting to fix the coronavirus as a mere object awaiting to be represented. No matter what position the representation takes toward the coronavirus and its significances, the will to truth turns a dedication quickly into a testimony and even a perjury.

A virus is an infectious agent that replicates only within a host organism. For the host, a coronavirus is a deadly stranger and an intimate family member at the same time. Familiar, frightening, and secretive, the coronavirus reminds us of the uncanny, as discussed by Freud. In his pioneering study, Freud focused on the unsettling psychological state of the uncanny. Distinct from the feeling of fear, the uncanny is a kind of terrifying feeling that is associated with something known and familiar. After an etymological investigation of the German words *heimliche*/*unheimliche*, and a close reading of Hoffmann’s story “The Sand-Man,” Freud (1964) unearthed the origins of the uncanny: “It may be true that the uncanny [*unheimlich*] is something which is secretly familiar [*heimlich-heimisch*], which has undergone repression and then returned from it, and that everything that is uncanny fulfills this condition” (p. 245). He also associates the feeling of the uncanny “with the omnipotence of thoughts, with the prompt fulfillment of wishes, with secret injurious powers and with the return of the dead” (Freud, 1964, p. 247). Following the lead of Freud, Derrida worked on the concept of the uncanny to engage with Marx’s concepts of repetition, specter, and fear. Refuting the claims that the tenants of Marxism have died, Derrida emphasizes the strange familiarity of the specter of Marxism in the age of advanced capitalism. As Derrida insists, the specter of Marxism will continue to return from the future to visit us, to live with us, and to alert us. Similarly, Derrida (2006) interprets the uncanny through the concept of absolute hospitality, in which “one may deem strange, strangely familiar and inhospitable at the same time (unheimlich, uncanny)” (p. 212). Remaining structurally open to future interpretation, the uncanny in Derrida’s account presupposes a materialism without substance, a messianic without messianism.

Derrida’s understanding of the uncanny is critical to my reading of the coronavirus as a ghostwriter of *envoi*. As a ghostwriter, the coronavirus is a ghost who writes from the future. As a stranger and a family member, it writes with and in the place of the host. By writing an *envoi*, a kind of writing haunted by failure and repetition, the coronavirus makes itself visible and frightening in a spectral moment. However, the *envoi* is not exclusively governed by a ghostly logic that is followed by and instantiated through the coronavirus. In critiquing the tendency to unearth an ultimate truth, Eve Kosofsky Sedgwick & Frank (2003) regard affects as a possible way out of the binary opposition of truth and falsehood in representation. By invoking the power of the performativity of shame, they highlight the negative affects neglected by identity politics, dismissed and stigmatized:

Without positive affect, there can be no shame: only a scene that offers you enjoyment or engages your interest can make you blush. Similarly, only something you thought might delight or satisfy can disgust. Both these affects produce bodily knowledges. (Sedgwick & Frank, 2003, p. 116)

In their view, shame is neither subversive nor mandatory; it works with other affects, drives, and representations to adapt the body to its situation. Foregrounding bodily knowledge acquired through trauma commits us to thinking differently about representation and the *envoi* in question. Fear of the coronavirus is not only the fear of a returning trauma as a ghostly logic in representation. More importantly, the coronavirus writes itself and writes about bodily memories of trauma in a constant play of materialization: inscribing fear in itself and on the body of the host permanently.

With the end of the lockdown in Hubei, the coronavirus pandemic is almost over in China. However, the coronavirus has been sending, and will keep sending, its fearful *envoi*.

## When the Virus Is Not Just a Virus: Nationalist Interpellation in a Global Pandemic (Srinivas Lankala)

The enduring sign of the Coronavirus pandemic for Indians was not related to medicine or public health. It was the unprecedented exodus of migrant workers from metropolitan centers to their native rural districts, sometimes hundreds of miles away (Mukhopadhyay & Naik, 2020; Petersen & Chaurasia, 2020). The scale of this migration was vast and is still being understood. It certainly provokes disturbing questions about urbanity and the fragility of a political compact that kept people in their place through calibrated deprivation (Dahdah et al., 2020). But for our purposes here, I will explore the ways in which it underscores the varying effects of the pandemic on different classes of people and the diversity of its signification.

The virus in India is both a medical event to be dealt with through appropriate public health measures and a mediated discourse that has developed its own ramifications and responses. I argue that both forms of the virus have had tragic and miserable consequences, but on different classes and groups of people. Like the televised Persian Gulf war of 1991 that Jean Baudrillard found to be a distinct and distorted signifier of the actual fighting on the ground,^1^ the virus itself is not the same phenomenon once it is transformed into a signifier for other meanings and purposes.

The novel coronavirus later named COVID-19 emerged in the public consciousness as a distinct problem with the rapid rise in infections in several Indian states by February 2020. In March, the Government of India mandated an immediate “lockdown” of the entire country. This new term burned itself into the national consciousness and its many vernaculars almost instantly, as its meaning became physically apparent. It involved the physical arrest of people wherever they happened to be at the moment, and the prohibition of all commerce, traffic, and circulation. It was announced with a 4-day notice period by the Prime Minister, in an eerie echo of a similar announcement in 2016 of the withdrawal of paper currency.^2^ That tragic farce had laid a historical precedent for this second tragedy to come. As a deeply iniquitous society and economy were forced to a halt, the effect was expectedly unequal. Metropolitan Indian citizens soon learned to cope with the new hardships of “work-from-home,” homeschooling, online classes and meetings, and such social-media-driven innovations as cooking and cleaning without domestic servants and entertaining themselves in their houses and apartments. The government also encouraged the adoption of derivative coping mechanisms as soon as they were observed in other countries: applauding medical workers from the safe confines of apartment balconies and terraces; singing, chanting and clanging metal plates and dishes with utensils in cacophonous, solidarity of the gated classes; lighting lamps and candles; and waving mobile phone flashlights at appointed times (Krishnan, 2020).

However, the actual effect of the virus became inseparable from the effect of the “lockdown.” The sudden impoverishment of the majority of the country’s population led to starvation, medical neglect, and a national panic. While invisible to the citizens in its first few weeks, it became impossible to ignore, when workers across Indian cities started to simply walk back to their native villages. Their exit from cities also emphasized the fragility of urban belonging: that in a crisis, Indian cities were fundamentally empty shells, drawing people not through cosmopolitan attractions or civic rewards but by rural misery.^3^ At this point, the virus was still largely a media phenomenon, while the “lockdown” was what had directly affected most Indians: The sudden disappearance of work, wages, commerce, and circulation magnified the precarity of urban existence. The largely informal national economy quickly unraveled in a crisis.

This crisis was exacerbated by the role of the virus in continuing the ideological and political discourses of the chaotic period immediately preceding the lockdown. The use of the virus to carry out “politics by other means” can be seen in other polities as well, but its entanglement with Indian politics is particularly useful as a means to understand the virus as a set of signifying practices. The context of this political use of the virus as a signifier is also inseparable from the highly mediatized nature of Indian politics and society.^4^

The virus emerged as a discursive phenomenon in India at a crucial juncture in a national conflict over changes to the country’s citizenship laws. With the rise to national power of the ruling Rashtriya Swayamsevak Sangh (RSS, or the National Volunteers Organization—a fascist group founded in 1922), India’s national government had been attempting since 2014 to achieve its political goal of abolishing its secular and liberal constitution through a steady dismantling of public institutions (Roy, 2020). This conflict worsened in 2019 with the re-election of the RSS-controlled government headed by the current Prime Minister, and the consequent repeal of laws that had hitherto guaranteed the autonomy of the occupied territory of Kashmir. This was followed by a critical change to citizenship laws to specifically exclude Muslims from gaining Indian citizenship and institute a new “citizens’ register” to determine afresh the legal status of all residents.^5^ With reports of the parallel construction of detention camps outside major cities, the fascist inspiration and ominous intent of the new laws became clearer and more immediate.^6^ Protests and political resistance to the new measures emerged across the country, and were met with violent responses from the police and RSS groups. Matters had reached a head when the nationwide lockdown was suddenly imposed.

Except in a few Indian states such as Kerala, with still functioning local health systems, the lockdown did not involve any public initiative to test or prevent the spread of the virus. Instead, in keeping with the ruling ideology of our time, citizens were mandated to protect themselves, on pain of being brutalized by the police if they failed. In this chaotic sauve qui peut scenario, the rhetoric of basic preventive measures took on ominous ideological connotations depending on who you were and where you lived. As it became clear that only access to clean running water, adequate space, and a home to live in would guarantee the efficacy of the public health guidelines, medical advice became meaningless for much of the country’s population, especially the inhabitants of vast informal urban settlements in the metropolitan cities. In effect, a dual situation emerged: A parallel virus had infected the classes who lived in gated urban communities and formal neighborhoods and who followed its progress in daily primetime news trackers. Positive cases, testing ratios, death rates, and other numbers soon flew across television and website screens in macabre charts, graphs, and complex animations, as breathless studio anchors enthusiastically tracked the competitive fatalities across states, regions, cities, and countries. As the formal state and civil society response to the virus grew more and more into a media discourse, its actual effect on the population was determined by existing social conditions and ideological practices than by the ideals of public health.^7^

In the early period of its spread, the illusion of its control was maintained through the interpellation of the mass television audience as ideal national subjects. In a series of televised speeches, the Prime Minister exhorted citizens to planned acts of mass discipline, such as the applause, noise-making, and lamp-lighting exercises mentioned earlier. It took several costly weeks for the citizens to realize that this national son-et-lumière had only served to deafen and obscure a different and more real virus that had silently spread illness and death among urban populations who did not have houses or apartments with balconies. A starved public health system soon proved inadequate and unprepared. Because this real crisis was not mediated or televised, there was no appropriate or meaningful response to it.

The easy congruity of the eagerly adopted virus prevention measures with the practice of caste-based rituals of discrimination was not lost on most Indians (George, 2020). This fortunate coincidence enabled the easy normalization of virus prevention as a legitimization of existing hierarchical practices. The convenient prescription of social distancing appeared to keep the privileged class of wealthy and respectably middle-class white-collar workers as far away as possible from the physical contact or proximity of their social inferiors. The pandemic thus seemed tailor-made for defenders of Hindu caste hierarchies, a righteous and suitably scientific legitimation of social discrimination.

The fantasy of caste purity would have remained an abhorrent social remnant if it had not become part of state policy in the last few years. But in the context of the state-led legitimation of religious hierarchies and the consequent onslaught on emancipatory laws, this entanglement of the virus with caste and with the violent hate crimes against Muslims acquired a dangerous dimension. It is this distrust of and disgust with a compromised public health system that drove so many Indians streaming out of cities and into the relative safety of their impoverished rural communities.

The alienation of Muslims as a national other has been a part of the basic doctrine of India’s current ruling group ever since its founders, awed by the Nazi policy of extermination, adopted a similar goal for the erasure of non-Hindu communities in India. The mass protests and popular uprising against the RSS’s attempts to irrevocably alter the basic structure of the country’s republican constitution had reached a tipping point when the COVID-19 epidemic was suddenly deemed emergent enough to impose an unprecedented “lockdown,” in effect a de facto police state across the country.

The imposition of the lockdown allowed police to destroy protest sites, detain protestors, and unleash a reign of terror across Indian cities. Caught in the initial crossfire were members of an apolitical Muslim religious group, the Tablighi Jamaat, whose convention in Delhi had been interrupted by the lockdown. Jamaat members trapped in the organization’s premises by the curfew were found to be infected with the virus. The consequent media narrative of the discovery of the infection among the Jamaatis veered into the fantastical, with nightly news anchors debating the strategies of a “corona jihad” that was to be waged by militant Muslims using the virus as a weapon (Perrigo, 2020). This dog-whistle narrative of Muslim bodies as unclean spreaders of a foreign disease dovetails with similar narrative frames used to portray Hindus from laboring and working castes as well.

The manufacture of conspiracies surrounding the coronavirus can be seen across the world and is not unique to India. A disturbingly large proportion of Americans, for example, appear to believe that the virus has been manufactured to enable mind-control through vaccination and 5G cellular signals by a ruling elite (Fisher, 2020). On rare occasions, these conspiracies do spiral out into real effects such as the bombing of cellular towers in Britain and the anti-vaccination movement in the United States. In India, however, the covert encouragement of such theories by the state itself, to legitimize the hatred toward Muslims, exacerbates and normalizes the rumors as mainstream prime time news which is then amplified and shared through an organized social media campaign (Ellis-Petersen & Rahman, 2020).

The vilification of the Muslim Other serves two purposes, one of furthering the state’s broader agenda of religious and caste purity, and the other more immediate goal of providing a scapegoat for the inescapable rise in infections and deaths due to the virus and the inability of the state and society to understand the crisis.

The brutal police crackdown that accompanied the lockdown and the violence of its imposition across the country were a small reminder of the routinization of the “lockdown” as a way of life in the occupied valley of Kashmir, part of the only Muslim-majority state in the Indian Union. The effects of the police state as a normalized entity have been multiplied since the abrogation in 2019 of constitutional laws guaranteeing the region’s autonomy, even if such laws were honored more in the breach in preceding decades (Zia, 2020). The uncanny resemblance of a public health curfew to a military occupation is not coincidental, but the result of the colonial origins of both, and of the state institutions they represent.

The symbiotic existence of caste-based discrimination, the extermination of a religious minority, and the colonial occupation of an entire province within the same body politic is made possible by the continuous interpellation of the mass of people to become national citizen-subjects. This call to obedience, broadcast daily through primetime television and magnified through the near-mandatory use of mobile phones,^8^ is the only sign of a nation-state that is otherwise absent in the real world. The failure to stop the spread of the real virus is obscured as the interpellated citizen is urged, cajoled, and threatened to participate in the simulacral fight against a mediated virus in a purely semiotic realm. The washing of hands without the precious reality of running water, the maintenance of “social distance” in the absence of space, the exhortation to “work from home” for a population that is not housed, and the discourses of online socialization and commerce are all much more than signs of mere denial: They are the components of this new semiotic space, enabling the call to national belonging in a new domain, bereft of its mooring in the world.

From a broader historical perspective, the coronavirus epidemic does not appear to have affected Indians as much as the far greater fatalities caused by more prosaic diseases, hunger, and the increasingly toxic air and water (Rukmini, 2020). What has caused the greatest pain and panic is the *response* to the epidemic. This response has been not to the virus itself, but to a simulacral virus that appears to occupy the same space and shares the same name as COVID-19, but which is a mere signifier, pointing to other, older evils. Like Baudrillard’s hyperreal war, it has surpassed the real virus itself and has come to occupy its place. It cannot be wished away or prevented with a vaccine, it needs a response in kind: of new counter-signs and counter-discourses.

## Reimagination of East Asia in the Chinese Public Discourse of COVID-19 (Yuan Gong)

A catastrophic pandemic unseen in a century, the current raging of COVID-19 around the globe has undoubtedly produced a unique symbolic site for global, regional, and national imaginations. As the earliest epicenter of this infectious illness, China has witnessed the proliferation of discourses about the evolution of the pandemic on various media platforms, through which the Chinese public has the rare chance to reflect on important issues regarding identity construction, social reformation, and nation building. While much attention has been paid to the stigmatization of China in Euro-American politics, media, and everyday whisper that label the natural coronavirus as a cultural and ethnic fault (Fu, 2020), what has been overlooked is how China has portrayed other countries in this global health crisis, especially those surrounding nation-states in the same geopolitical area. East Asia, or the Sinosphere in the broader sense, with the collective memory of fighting SARS in 2003, is thought to have responded to COVID-19 more efficiently than many Western countries (Salmon, 2020). How, then, is the East Asian encounter with COVID-19 depicted in the Chinese public discourse? How does such depiction envisage China’s relations with neighboring countries and its position in the area?

In this essay, I discuss the ways in which the coronavirus pandemic has been appropriated by the Chinese public for a (re)imagination of East Asia. By exploring the evolving representations of its neighboring countries throughout the epidemic on Chinese media platforms including Weibo, WeChat, and Zhihu, I argue that the talk of the regional responses to COVID-19 envisions a China-centered union of selected East Asian countries in parallel with the historical tributary system of the Sinosphere. Through the expression of the nostalgia for Imperial China, the discursive reconstruction of the East Asian identity is a ratification of China’s contemporary ambition to reclaim its geopolitical dominance.

### Mutual Support for Regional Crisis

Synchronized with the rapid transmission of the coronavirus in China and East Asia between January and March 2020, the Chinese public in this early phase drew close attention to the unfolding of the epidemic in its nearby countries, and Japan and South Korea in particular. With the disease breakout involving Diamond Princess (Japan) and Shincheonji Church of Jesus (South Korea) frequently making news headlines, the discussions of how those countries responded to COVID-19 flourished online, which, in combination with the continuous debates over China’s own pandemic threat management, contributed to the imagination of the COVID-19 rampancy as a regional challenge that China and its neighbors faced together.

Central to the discursive formation of this imagined community was the celebration of the incessant interaction and cooperation between China and some East Asian countries to combat the virus collectively. In the wake of the outbreak when China was threatened by the crumbling of its health care system, the countries under the spotlight—Japan and South Korea—were widely appraised for the sympathetic and supportive approaches they took to help China overcome the severe shortage of medical resources. The media reports of Japanese and South Korean governments leading the international aids to China (Gong, 2020) were echoed by numerous warm anecdotes on social media championing the heartfelt support from their people. Perhaps the most well-known story of this kind, a Japanese institution wrote a Chinese-language verse on the boxes of masks it donated to the province of Hubei: “Rivers low, mountains high; The same moon in the sky” (trans. Zhao, 2020) (“山川异域，风月同天”), which immediately went viral online because of its signification of the long-lasting friendship between China and Japan. According to *Account of the expedition to the east by the Great Master* (唐大和上东征传) written by Omi no Mifune (淡海三船) (see Wong, 2018), this sentence was from an ancient poem written on the edges of the Buddhist robes Japanese missions (遣唐使) brought to Tang China as the tribute from Prince Nagaya (長屋王). Given its profound roots in the history of Japanese envoys to Imperial China learning from the Chinese culture and civilization, this verse went beyond re-fostering the traditional Sino-Japanese solidarity. Analogizing Japan’s mask donation with ancient Japanese envoys’ gifts, it also evoked the retrospective commemoration of the hierarchy between China and Japan in history which almost vanishes in the modern era. Therefore, the popularity of this verse may indicate the aspiration for the reoccurrence of such bi-lateral relations.

Indeed, this was only one example of the ubiquitous imaginary of the pan-East Asian cooperation and exchange of goods and information as a modern emulation of the tributary system through which Imperial China maintained its diplomatic and trade relations to neighboring countries and consolidated its dominance in the region for over a millennium. After China started to keep the pandemic under control and resume the production of medical supplies, this metaphor was further perpetuated in an attempt to accentuate that China’s supplies of medical goods and anti-epidemic lessons to nearby countries drastically outnumbered what it was initially given. On Weibo, China’s return of masks and respirators to its neighbors was often explicitly compared with the “vassals’ gifts” Chinese emperors assigned to tributary states in posts like this:

Tribute is both the highest form of alliance and an advanced way of investment, but (this time) it is based on masks! Recently, Xinwu District in Wuxi, Jiangsu Province donated 50,000 to Toyokawa, Aichi Prefecture in Japan in return for the 4,500 masks, protective clothing and other anti-epidemic materials Toyokawa donated to Xinwu District in February. (Weibo source, March 25, 2020)

This nostalgic use of metaphor implies a crucial undertone of Sinocentrism of the public imagination of the community comprising China and bordering countries fighting against the coronavirus. The tracing of the origin of East Asian solidarity to the past is suggestive of the ambition of the present. The Chinese public not only fantasizes about a reunion of China, Japan, and South Korea for COVID-19 but more importantly yearns for the recovery of their nation’s leadership and centrality in this battle.

### Victory of the “Confucius East”

As the coronavirus expands rampantly to the rest of the world from March 2020 onward, Chinese media coverage quickly catches up with the shift of the epicenters from East Asia to Europe and North America and reformulates the pandemic as a global health crisis. Against the depiction of how COVID-19 created chaos, helplessness, and dysfunction in Western societies stands the stark contrast of East Asia as a safer zone where the outbreaks have been largely contained with success. With the similar control of cases less than 20,000, Japan and South Korea remain at the heart of this imagined safe zone in company with China even though the reality has seen even fewer confirmed cases in other parts of Asia as well as the recent resurgence of virus spreading in all these three countries.

This rhetoric is in concert with the prevalence of online deliberations about why East Asia as an area has performed better than other parts of the globe in the containment of the virus. At the core of these discourses lies the construction of an East/West binary which frames the global responses to the pandemic into a competition in which “We” (the East/East Asia) have triumphed “Them” (the West/Euro-America). Although China and neighboring countries diverge in the official approaches to handle the pandemic, their relative efficiency in virus containment in comparison with the West is considered to be guided uniformly by the cultural values they share as part of the “Confucius East.” In particular, collectivism—the principles of prioritizing community interests to personal interests, pursuing social harmony, compliance to authority, avoid causing inconvenience to others—has been glorified as the main drive for the people in East Asia to more effectively cope with the governmental strategies in contact tracing, testing, social distancing, and mask wearing. Similarly, the regional cooperation in the pandemic management is regarded as a manifestation of these values. For example, the reflections on how South Korea has set a model of disease control using mass tracing and testing tend to recognize the smooth uptake of this procedure facilitated by Koreans’ collectivist mind-set that downplays individual privacy and complies with the data-mining measures to track and publicize their locations, activities, and close contacts. Meanwhile, other popular discussions blame the religiosity of the Shincheonji Church members whose gatherings caused the initial COVID outbreak in South Korea, which is reflected from the titles of Zhihu posts that describe the diffusion of the virus through “Hallelujah” such as “The occupation of South Korea by Covid-19, everything has to start from ‘Hallealia’” and “South Korean cult Hallelujah devastated the country.”

Apparently, these titles have no intention to mask the underlying tone mocking at the role of Christianity in the acceleration, not mitigation, of disease spreading, which further serves as a foil to the power of Confucianism to help South Korea navigate away from the disaster. In fact, satire targeting at Christianity represents the broader criticism of Western cultural values in hindering the efficacious enforcement of restrictive and surveilling measures against the coronavirus. The East Asian identity is thus reaffirmed through the clashes between the Eastern and Western civilizations. However, it is worth noting that the narratives about the East Asian conquest of COVID-19 are again permeated with the metaphor of the tributary system delineating China as the leader and role model in this imagined “safe zone.” Not only does the attribution of the regional success to Confucianism call up the historical Chinese centrality in the Sinosphere but the emphasis on China’s ability to offer lessons and instructions from its early experience for its neighbors to benefit from also ratifies the restoration of the “teacher/student” relation between Imperial China and pre-modern Japan and Korea.

### A Process of Inclusion and Exclusion

Far from a total reenactment of the historical Sinosphere, this Chinese imaginary of East Asia engages with a purposeful selective process that amplifies China’s solidarity with some East Asian countries but simultaneously mutes others in the same region. As remarked earlier, a majority of the online narratives about the cooperative responses to COVID-19 in East Asia revolves around China, Japan, and South Korea, with less frequent inclusion of Singapore as well as occasional reference to such countries as Mongolia and Myanmar. This emphasis on forming a coalition with Japan and South Korea is compatible with China’s long-term agenda of promoting and dominating the China–Japan–South Korea union (中日韩一体化), which was recently reiterated by the three governments’ consensus to speed up the negotiation of the free trade zone (中日韩自贸区) (Wang, 2019). In this sense, the COVID-19 crisis has offered a discursive site for the Chinese state to rebuild this trilateral bond and remodel its significant neighbors whose national images, due to the respective disputes around Diaoyu Islands and THAAD (Terminal High Altitude Area Defense), have been negative in China for almost a decade.

While the China–Japan–South Korea triangle is romanticized in connection with other small countries, the alienation of some Confucius societies from this imagined “cooperative” East Asia is quite striking, especially given the outstanding results some of them have produced in the prevention of disease transmission. The first excluded category includes Taiwan, Hong Kong, and Macau—the territories outside the mainland in the Great China area. Whereas Macau is often forgotten by the media as it has always been, both Taiwan and Hong Kong are widely criticized and mocked for their attempt to politicize the pandemic as a weapon to confront Beijing and increase international recognition. The second group pertains to North Korea and Vietnam—the authoritarian states that have close political and ideological bonds with China. For instance, North Korea has been constantly questioned and satirized because of the lack of transparency in the disclosure of its epidemic circumstances. Vietnam’s outstanding handling of the virus which led to only 334 confirmed cases and 0 death was nearly silenced in the mainstream media coverage. In the unusual reference to Vietnam in some Zhihu conversations, Vietnam’s success was rarely celebrated but considered as a “threat” to China’s leadership in containing the pandemic in the area.

The trivialization and exclusion of these countries/regions from the Chinese imagination of East Asia as a collective force fighting against COVID-19 is not unexpected. In the first place, the negative attitudes toward them (except Macau) reflects a backlash against the restrictive, non-cooperative methods those governments have enforced to block the virus from mainland China (e.g., full border closure; ban on exports of medical supplies), which signifies their resistance to be incorporated into the modern tributary system Chinese people have aspired. Yet for Taiwan and Hong Kong, this exclusion repeats the endeavor of Chinese official propaganda to erase the distinction between them and the mainland and disavow their political autonomy. Instead of being completely out of the picture, their responses to the coronavirus are mainly discussed as part of the Chinese experience to consolidate the national identity. For North Korea and Vietnam, the negative impression may partly result from China’s ongoing diplomatic conflicts with them in recent years regarding the South China Sea and denuclearization, respectively. Nevertheless, the shaking of the “socialist brotherhood” on the matter of COVID-19 also implies the reluctance of the Chinese public to articulate a regional identity around the axis of a shared political regime. In fact, assimilating itself with ideological and political allies is likely to obscure the focus of this imaginary on China’s historical and cultural alignment with Japan and South Korea.

### Epilogue

As COVID-19 begins to shift both the scholarly and media focus on an international scale to reconsidering the dark sides of globalization (Chan & Haines, 2020) and mourning for the disruption of European Union (Trofimov & Pancevski, 2020), China’s reversed agenda of imagining a regional union is stunningly intriguing. On one hand, the eagerness to build solidarity with East Asian countries represented by Japan and South Korea might be a strategy to react to the racialization of COVID-19 as a “Chinese virus” and the demonization of China as a “public enemy” and “trouble maker” in the Euro-American political and media agenda (Viala-Gaudefroy & Lindaman, 2020). By articulating China’s resemblance (and collaboration) with the bordering democratic capitalist states (rather than the “socialist brothers”) in the “Confucius-inspired” success of halting the virus, the public discourse strives to construct a collective identity of the East so as to brush off China’s label of the Other imposed by the Western imagination. Ironically, this consolidation of the Eastern identity also serves as a repercussion to otherize the West as the loser to the coronavirus.

On the other hand, the rise of this East Asian imaginary centering around China’s historical and cultural bonds with Japan and South Korea has far-reaching implications for China’s geopolitical strategies beyond the COVID-19 pandemic and the realm of public health. Rested upon the trope of the imperial tributary system, this imagination reflects how the Chinese public discourse echoes the state ambition to recuperate the historical dominance of China in the Sinosphere, which is part of Chinese Communist Party’s long-term project of “the great revival of the Chinese nation” (中华民族伟大复兴), or in Xi Jinping’s term, the “Chinese Dream” (中国梦). Incorporating Japan and South Korea—the most important American allies in East Asia—into the imagined tributary network might serve the specific purpose of weakening the U.S. hegemony in the region (see Ikenberry, 2004), whereas the tactic exclusion of North Korea and Vietnam indicates the indifference of many Chinese to the state’s political and ideological “comrades” (whose traditional alliance with China has often proven itself unstable and delusionary in the changeable economic and political dynamics in East Asia). More importantly, this selective reimagination of the Eastern union expresses the Chinese public’s nostalgic ideal of the nation’s revival, which dreams of a return to the Middle Kingdom, the empire that reunites and leads East Asia through culture and history.

## Making Viruses Matter (Xuefeng Feng)

### The Toxic Present

During the year 2020, which is anticipated to be the warmest year in human history, we failed to stop the rampant spread of a coronavirus called COVID-19 and its disastrous impact on societies and individual lives. Unlike its “cousin” SARS, which broke out in early 2003 and vanished into thin air largely because of rising temperatures, the current respiratory epidemic has yet to show any sign of amelioration with the arrival of summer.

News photos have shown audiences an incredibly bleak, bizarre, and somewhat surreal picture of life during the pandemic. Streets are evacuated. Stores are closed. Public services are paralyzed. Modernized cities have become empty and ghostly quiet. Only scattered people equipped with medical face masks walk anxiously in this futurist, apocalyptic scene.

To use Timothy Morton’s concept, the COVID-19 pandemic has become a “hyperobject,” a phenomenon that possesses an ahuman time scale and an extremely diffused quality in occupying space. In such a space–time reconfiguration, or, in plain language, during this type of disaster, humankind becomes an obsolete idea, as humans no longer play a meaningful role in the space–times created by and for “hyperobjects.” Unfortunately, such a concept bares relevance in light of the uncontrollable proliferation of the coronavirus across the globe at this juncture.

Worse still, some epidemiologists warn that a new round of outbreak will likely occur soon in the coming fall. A possible scenario could repeat the conditions after the 3/11 Fukushima Daiichi nuclear disaster, when breathing with face masks, people eventually became accustomed to a state of emergency as the conditions for living and dying in the Anthropocene. “[P]oison has become a normal feature of daily life, the second nature we have to inhabit” (Berardi, 2012, p. 12). While one can attribute the deterioration of nature to neoliberalism and its disastrous governance, this essay, rather, speculates on what foregrounds the involutional relationship between humans and the earth beyond the “nature-culture” divide. Whether one is willing to admit or not, viruses are neither creation ex nihilo nor culturally and politically constructed representation. Instead, they are beings that have always been part of earth’s composition.

### Neither Object nor Subject

In a prophetic book, *The Natural Contract*, the late Philosopher Michel Serres (1995) describes the evolution of the earth’s composition. In ancient law and modern science, nature was treated as an objective reference point, because it had no subject. Existing objectively “out there,” the earth was a space that did not depend on humans but only acted passively in relation to causality. Yet, witnessing the ecological crisis arising in the 20th century, humans realized that the earth has been affected by our behavior and is now behaving like an aberrant subject! In recent scholarship, this subject has been referred to as Gaia, the capricious goddess of the earth (see Latour, 2014, p. 3).

The earth is full of action and so is COVID-19. As described in news reports, the coronavirus looks for and hijacks its hosts; it finds easy purchase on, and takes control of, human bodies; it kills many, but not all, of its hosts so as to keep moving, spreading, replicating, and surviving. It would be impossible to talk about the virus without referring to those actions. Cited by the *Washington Post*, a virologist came up with a vivid analogy for viruses by comparing them with destructive burglars. “They break into your home, eat your food, use your furniture and have 10,000 babies” (Kaplan et al., 2020).

As the word “object” refers to entities that are inanimate and subject to chains of causality, viruses, in this sense, hardly fit into this definition. For instance, COVID-19 remains mostly enigmatic, not least because it is considered strikingly sneaky—“the virus doesn’t really want to kill us. It’s good for them, good for their population, if you’re walking around being perfectly healthy,” said another virologist in the same *Washington Post* article (Kaplan et al., 2020). Besides doing things such as breaking-into, eating, and having-babies, the virus is further endowed with intentions—it does not want to kill us!

However, the coronavirus should not be mistaken for a subject, especially a subject–agent, which is historically associated with liberal humanism since the Enlightenment and which is deeply rooted in the “nature-culture” divide, an ontological regime referred to by Latour as “the Modern Constitution” (see Latour, 1993b). The idea of the subject as a product of Euro-American modernity is indivisible from its aim to achieve individual sovereignty and autonomy. In a politico-legal sense, bounded individualism is the most evolved form of this idea in the wake of the global expansion of capitalism. Faced with an unprecedentedly active earth in the late 20th century, nonetheless, this anthropocentric conception of the subject–agent has been confronting exponential challenges, among which the current coronavirus pandemic constitutes the latest one. To be clear, the term “subject” is a mismatch for COVID-19, not because it is agentless and incapable of doing the same things that humankind does. The contrary is true: The state of being of the virus—what it is—can unfold only through its actions and long after its performances. At stake for the virus and humans is that there are “no pre-constituted subjects and objects, and no single sources, unitary actors, or final ends” (Haraway, 2003, p. 6) Far from being a de-animated object, or an anthropomorphized subject, Gaia, the increasingly “rioting” earth, is a collective of actions that distributes agency in heterogeneous and surprising ways. As a result, “we must not believe in advance that we know whether we are talking about subjects or objects, men or gods, animals, atoms, or texts” (Latour, 1993a, p. 167), and also viruses until their actions are captured, and rendered into shapes—whether the shape of a human or of a virus.

### Staying With the Trouble!

The story of the human-centered history is being replaced by an explosion of narratives about the increasingly animated and animating earth. However, the dualism of the subject versus the object, unfortunately, is still perniciously conserved in the mainstream reaction to the COVID-19 pandemic. When societies are forced to act on the pandemic, the virus is almost exclusively treated as an object subject to the chain of causality.

This tendency is clearly reflected in the mobilization of wartime rhetoric and discourses in conjunction with governments’ anti-epidemic measures. For instance, when visiting Wuhan right after its lockdown, Sun Chunlan, China’s Vice Premier, warned that the country was facing “wartime conditions.” Likewise, only 1 month later, President Donald Trump declared a national state of emergency over the coronavirus outbreak in the United States. In this antagonistic discourse, contending with the virus, a not-yet-tamed and potentially threatening other, is framed as a relationship between humans and their enemies. For those who believe humans and only humans make history, a self-proclaimed war on the virus is unavoidable! Peace, accordingly, is only imaginable to be reached, or more precisely restored, to an already existing order, established primarily for humans.

Mobilized to describe the relationship between COVID-19 and humans, “war” is a terrible and even dangerous choice in terminology, due to its undertone of human exceptionalism. In fact, nearly 90% of the cells in a human body is “part of a vast community of companion species, particularly bacteria and viruses” (Smart & Smart, 2017, kindle 72). Unfortunately, most humans have yet to learn the meaning of living and becoming-with these beings who are made by and making humans at the same time.

In her book *Staying With the Trouble: Making Kin in the Chthulucene*, Donna Haraway (2016) invites readers to contemplate our troubling present, the Chthulucene, an emerging regime of naturecultures, as opposed to the “nature-culture” divide. Contrasting to the discourse of the Anthropocene and the Capitalocene, both of which are conceived as human-induced condition, the “Chthulucene” is, first and foremost, concerned with earth beings who live in “manifold forms and manifold names in all the airs, waters, and places of earth”—they are monsters which “demonstrate and perform the material meaningfulness of earth processes” (Haraway, 2016, p. 2). The vicious coronavirus is evidently one of these monsters. Despite the havoc it is creating in the present, the epidemic is a manifestation of the biotic and abiotic powers inherent in earthly actors and is part of “ongoing multispecies stories . . . in times that remain at stake” (Haraway, 2016, p. 55). As implied by the title, one of the valuable lessons of Haraway’s book is that, for humans in particular, there might be no better option other than to stay with troubles, of which humans are never innocent.

Staying with the troubles demands caring for all the threads that bind us together and make our existence possible in the first place—humans are made by countless earth beings and vice versa. It also means that we are required to weave unexpected and even dangerous connections with others, in Haraway’s (2016) words, making kin as oddkin “in unexpected collaborations and combinations . . . We become-with each other or not at all” (p. 4). This insight is particularly useful for thinking about viruses. Because viruses have “no cellular machinery of their own, they become intertwined with ours. Their proteins are our proteins” (Kaplan et al., 2020). In this sense, the evolution of humans and viruses is inseparable from the process of involution of the two into one. In other words, becoming-with means that, by definition, a “we” always precedes an “I,” a “you,” or a “they.”

The so-called “asymptomatics” provide an excellent example of this point. Asymptomatics refer to those who test positive for COVID-19 but, confusingly, do not suffer from illness or show any symptom of the disease. Asymptomatic infections or carriers are possibly greater in number than those with symptoms. At this point, it is impossible to decide which of the two types is more typical of COVID-19 infections, because, as a researcher at the University of Oxford says, “there is not a single reliable study to determine the number of asymptomatics” (Shukman, 2020). In the same news report, Neil Hall, a biomedical expert, suggests considering asymptomatic cases of the coronavirus as the “dark matter” of the epidemic, as invisible and not-yet identified dark matter is believed to make up most of the matter in the universe.

Despite the fact that no conclusion has been reached about the enigmatic phenomenon of asymptomatics, the differences that manifest among patients reveal that the virus, and the particular cases of infection, should be examined as specific units. In other words, between the virus and humans, the specificity of an encounter matters. Unlikely to be autopoietic systems that reproduce autonomous units, the virus and an infected body constitute a collectively produced, sympoietic system that does not have self-defined spatial or temporal boundaries. In these cases, and from a non-anthropocentric, philosophical point of view, the idea of bounded individualism has to be discarded for good. Beyond the divide between the subject and the object, what emerges are ontologically heterogeneous practitioners who are involved in each other’s lives. Besides evolution, living also relies on involution.

Without any intention of “romanticizing” COVID-19 and the current pandemic, staying with the trouble, as articulated by Haraway (2016), is “to make kin in lines of inventive connection as a practice of learning to live and die well with each other in a thick present” (p. 1).

### Mattering Viruses

The coronavirus does not happen as a matter of fact, which “passively” waits to be discovered, investigated, tamed, or neutralized by “active” humans. What we call the COVID-19 pandemic manifests itself as a differentiating and relational effect because it matters by bringing into being various relations between humans, and between humans and their oddkin. In this view, science is only one practice among many others to capture the efficacy of its mattering. In addition to biomedical measures, a more critical question for the coronavirus crisis is “what method does the matter demand” (Thompson, 2018, p. 13)? Proposed by Haraway for living in the Chthulucene, the string figure might also serve as an appropriate method and image for the pandemic, characterized by its exceptional contagiousness and interactivity. Consisting of “passing on and receiving, making and unmaking, picking up threads and dropping them,” the string figure is all about “becoming-with each other in surprising relays” (Haraway, 2016, p. 3). Crucial to this method is that it does not guarantee what is obtained turns out to be good in the end, because living itself has become so dangerous in this very thick present—agencies are distributed, conflicting, and entangled in a myriad of practitioners, human and non-human alike.

In this pandemic, we are all playing the game of string figures with our oddkin. It is not beneficial to judge in advance who is a subject and who is an object, or which one is active and which one passive, as all participants might be capable of something that matters in one way or another. For example, one thing that the respiratory disease teaches us is that not only breathing matters but also the manner how one breathes matters to others. Life and death happen inside specific connections and their mattering in mundane, and even fleeting, encounters. Making COVID-19 matter requires us to reanimate “what is coming into states of matter and mattering in bodies, stories, acts, and events” (Stewart, 2018, p. 24), in other words, in the vicissitudes of our ordinary lives. For the future of this thick present, one key is to stop imagining the crisis of the coronavirus as something wholly predicated on effective vaccines and scientific solutions. Instead, humans must learn to connect and also care for threads, some of which are obvious, some elusive, some vicious and dangerous, and some fictional. We may need to discard terms such as “overcoming” or “solution,” and turn to terms like “participation” concerning all that we are uncertain of but have to live and become with, together, in the “metamorphic zone” called the earth (Latour, 2014, p. 13).

## Of Viruses and Men (Briankle G. Chang)

What does it mean, the plague? It is life, that is all.

—Albert Camus, *The Plague*

The most abundant biological entities on Earth, viruses are forever and everywhere. Suspended between living and being dead, they are simply there, a slimy strip of ribonucleic acid (RNA), as biologists tell us. Poorer in life than tardigrades, incapable of movement, and having no logistic of their own, they ride on and feed off others to replicate themselves, to become the viruses that they are. As smart schoolchildren know, they are transmissible and must be so transmitted as to go viral, to become the viruses as we know them.

### Viral Media

Dependent entirely on carriers, that is, exploiting others’ hospitality, without which they have no life (but also no death either), viruses exemplify transmissibility. They live and thrive, as it were, only if their hosts are susceptible, in motion, and in contact and they die or die down when susceptible hosts are either unavailable or no longer hospitable. *De*fined, that is, made finite, by transmissibility, and yet transcending its barren finitude through parasitism, viruses exist and operate like pure media, self-generating and self-generated by being entirely coterminous with the channel through which they flow and multiply. Interpolating and encoding themselves in the metabolic cycle of others, thereby reproducing themselves passive-actively, they mediate by colonizing others and, in so doing, mediate themselves by proxy, going about so energetically and indiscriminately as to cause the demise and thus thwarting unwittingly their own propagation. If viruses communicate anything, if their shadowy occupation of host bodies sends any message, it is their very own communicability, their ability to disseminate themselves over a large population with effort less than minimal.

Although all over creation and in abundance, most of the viruses cause us no harm and we pay them little attention, even though they populate our body and capitalize on its resources. They become a matter of grave concern when they *infect* us, when they not only put themselves inside (*in-ficere*) our body and stain its normal functioning, but also threaten to afflict as many people as their “infectivity” attacks. More dangerous and less tamable than most microbes, viruses invade our body and compromise it at the cellular level. They do not just make us sick; they bring about plague.

Once seen as a cause of infection, viruses accrue significance and take on the label “pathogens.” To refer to viruses as pathogens implies that they are “medicalized,” that they not only enter into a relation with humans who regard them as toxic and virulent, but are also seen as a problem to be addressed in a methodical, systematic, that is, “scientific,” manner. It is through this medicalization that viruses are individuated and identified as a distinct biological entity and, having been so captured and given a name—for example, H1N1, Mers-CoV, SARS-CoV-2, COVID-19, and the like—by what might be called the “clinical gaze” and its taxonomic procedures, they enter into sciences and become a focus of medical research, made all the more pressing if and when they create public health crisis. More than one hundred years after Martinus Beijerinck gave the name *contagium virum fluidum* (contagious living fluid) to the incitant of Tobacco mosaic first discovered by Adolf Mayer and Dimitri Ivanovsky, viruses are now actively collected, classified, and manipulated by scientists in highly restricted spaces called laboratories, most of which, like the viruses housed carefully therein, are hidden from public eyes. While slimy poisons were once thought to be sent down by God to punish us for our sins, we now see viruses not only as an object of scientific investigation but also as a medical challenge that nature poses to us as biological creatures on Earth. Like birds, bats, and rats, we are all equal opportunity hosts to killer germs.

### Pandemics, *Unde Venistis*?

Not all viruses are fully pathogenic, but pathogenic viruses are ever ready to go viral when the conditions are ripe. However, although viral infection may break out and spill over, it does not mean that there is a pandemic. “Pandemics,” as virologists tell us, “begin when a brand-new virus infects a human who also at that point is able to transmit the virus to other humans” (Buettner, 2020). Two points should be noted without delay. First, pandemics are not created by transmission of viruses from some source *to* humans, but from humans *to* humans. Breakouts of viral infections among members of a primate community deep in the Amazon rainforests, for example, may be large scale and may disturb ecological balance alarming to conservationists, but they do not for all that count as pandemics in the sense that the term is properly used. Viruses might infect one or more individuals, but humans are responsible for creating the conditions that transform infections to outbreaks and outbreaks into pandemics. Pandemics, in other words, are not natural or biological phenomena; they name a human crisis, a contagious malady plaguing humans who are both agents and patients at the same time. Contagious diseases are disastrous to all, locked, as we are, in the same bubble in which microbes live and grow, but pandemics are decidedly more pernicious in that we become, often unknowingly, the source and the cause of our own infestation.

Second, pandemics are “declared.” As is the case with catastrophic events in history, like wars, famines, or mass cultural anomaly as bizarre as the Chinese Sorcery Scare of 1768, whose duration and identity result from an act of punctuation and sense-making entirely sociopolitical in nature, pandemics too begins with a performative act that announces their beginning and, having made them to begin in this way, determines when they reach their end, even though the viruses and their carriers may still be with(in) us (see Kuhn, 1992). Naming not microbial activities in nature but a crisis for humans, pandemics are events made real, public, and urgent, as just said, by a performative—a speech act, to be exact—whose authority in pronouncing their beginning and end depends on the very force that makes the declaration authoritative and forceful in the first place.

Brought into being by discourse and public communications, pandemics are social constructions; they signal a state of emergency—appearing, first, as physical ailments on the part of individuals, subsequently identified and ratified by medical and scientific community as a real health problem, and finally materialized by authoritative broadcast and public acknowledgment, thereupon becoming a public policy issue to be addressed by political leadership, all these over a determinate territory. Once established as such, a pandemic individualizes a collective paroxysm, making it a public enemy by giving it a face, a name, a certain life span in the social calendar, without which the havocs wreaked by the virus would not be the crisis its name designates and invokes. It is in this declarative nature of pandemics that we can see how viruses, once medicalized and publicly acknowledged, are inevitably entangled with science, history, culture, and politics. Socially constructed by a *de*cision, by a cut or break into regular time, they mark a “zone of exception,” a temporal heterotopic, as it were, where we, individually and collectively, stand to one another as equal subjects to illness, unfreedom, and death in the unending drama of man against nature and its hostile elements.

Viruses are viruses are viruses. They have no political content; operating according to the laws of physics, chemistry, and biology, they come and go on their own rules and on their own times, as nature dictates. In sharp contrast, pandemics are biopolitical phenomena; they are moments of discontinuity or rupture in social order, shot through from start to finish with forces and factors that shape culture, history, and economy, which in turn determine what they mean and how they come about and come to pass. Moreover, and importantly, a pandemic is not a single, monolithic event; it is a series of localized epidemics, each with its own point of origin, its own history, its own epidemiological pattern and impacts. Further still, all these factors crisscross one another in a complex, nonlinear fashion, amassing multiple agents and stakeholders in such a critical fashion that the language of war is often used by the authority in charge to quell the infectious assault.^9^ Pandemics force social changes precisely because the changes they incur invite resistance. It is for this reason, perhaps for this reason alone, that pandemics inevitably appear as a site of social contestations, politicizing and politicized by the heterogeneous constructions barely betrayed by the name of a single virus.^10^ It is for this reason too that pandemics assert themselves as a sign of generalized cultural and economic strife, a symptom of social struggle underlying the *health terror* that a viral breakout unfailingly induces.

COVID-19 is a novel virus, novel in that scientists do not fully understand how it afflicts the body and therefore cannot predict its epidemiological paths. To control its spread, we have no choice but to employ methods developed from past experiences, such as quarantine/isolation, social distancing, face coverings, and contact tracing, to name a few now well-known. Because viruses are infectious, to control its spread is, understandably, to separate and to isolate. This means that people be kept away from one another. Instead of gathering or being together, we make ourselves scare; better yet, we isolate ourselves, even if begrudgingly. More than that, the injunction of *isolation* leads straightaway to *insulation* in that the ultimate, foolproof means of isolation is to literally atomize ourselves, to turn ourselves into windowless monads. Indeed, all the mitigation measures we hear about of late—quarantine, mask wearing, hand washing, and social distancing—are in reality *anti-social* measures. Don’t reach out and don’t touch anyone! Cover up your face! Just as *social* distancing—a contradiction in terms of sorts—means keeping *physical* distance, and just as mask wearing reduces mutual recognition based on simple vision to its unnatural minimum, (self-)isolation and quarantine all but eliminate human contact of all kinds. When the plagues struck, we were all lepers; when COVID-19 strikes, we are all windowless monads. Pandemics are born of communicable diseases, yet for this reason, they force us to be incommunicable. Flattening individuals and bringing to a halt exchange and commerce of every kind, they turn a society into one that is *against* society. If there is a history of pandemics, it is a history of anti-social history.

Neither alive nor dead, neither this nor that, viruses are by nature improper. Never proper, that is, never being (of) themselves, they appropriate—always ready to make others their own. They are pure media, as suggested earlier. Viruses are pure because they mediate unconditionally. However, inasmuch as unconditional mediation performed by viruses leads to the demise of their host, upon whom they depend for their parasitic reproduction, viruses end up annihilating themselves by their very nature; they are always already their own collateral casualties. Rendering themselves nil by simply being and subsisting as themselves, pure media are no (longer) media. Unconditional mediation ends all mediations. By bringing society to go against itself, viruses commit suicide, so to speak, by killing their host, by the unconditional abuse of others’ hospitality. And, alas, we—at least some of us—are spared.

### Normal Returns?

COVID-19 is a new virus. But, unlike the known flu viruses, or H1N1, SARS, and the like, COVID-19 is considered “novel,” not the least because, as indicated earlier, it frustrates scientists’ understanding. “It has been like nothing else on Earth,” says an infectious-disease expert, who falls victim to the virus; “I knew I had the disease; it couldn’t have been anything else,” but “I don’t understand what’s happening in my body” (Yong, 2020). There are many things, inanimate or living, on Earth that are like nothing we know so far, and there are many things happing in our body that we do not understand at all. COVID-19 can justifiably be called “novel,” but isn’t every virus novel in its own way and at some moment in time? Isn’t being novel the normal course of event in life and in life sciences as well? “There is novelty here,” remarks a prominent epidemiologist Karl Friston upon leaving a lab meeting about COVID-19, but he quickly adds, “so this, from my point of view, is just an average day” (Kosner, 2020). Being novel is the very characteristic of all viruses and many other things in nature as well. The novelty of COVID-19 may not be as novel as we think.

What is possibly novel about COVID-19 is the fact that it gives us the first pandemic in our truly globalized age. The *global village*, in which we now live, is so hyper-connected—not only by technology but also through affluence, commerce, and global travel—that an infectant can travel from one city to another as fast as jet streams flow. Connectivity translates qualitative diversity into measurable multiplicity, reducing distance and difference for the formation of the common, which in turn strengthens connectivity. To be alive, as few would disagree, is to be connected, literally and in every other sense. But this means that we must live in and with the risks that global connectivity brings to us. To be connected brings with it the possibility of being stranded in harm’s way. As COVID-19 makes clear, “connectivity is the killer” (Kosner, 2020). After all, life depends on maintaining boundaries and keeping differences. Deadly viruses are deadly because they breach them.

As infection rate rises, so does anxiety. And bleak scenes spread as wide as the virus goes. Deserted streets, boarded-up stores, closed factories, shot-down public transportations; remote learning, work-from-home; stock markets crashed . . . and, worse yet, “I just lost my job.” Individual solation leads quickly to desolation across the board. And economy bears the brunt of a colossal coronal attack. Shortly after COVID-19 spread out of Wuhan, China, to Europe in January 2020, stories about the economic plight began to top the list of topics in public forums and news media. The future we face seems to lie in one of the two choices: to die from hunger or to die from the disease (餓死或病死), as the expressions go in Chinese media. It is not for no reason that a policy brief released in June 2020 by the United Nations on the impact of the pandemic is given the title “The World of Work Cannot and Should Not Look the Same After This Crisis” (Guterres, 2020). The address on the launch of this brief, given by the Secretary-General António Guterres (2020), begins as follows:

The Covid-19 pandemic has turned the world of work upside down. Every worker, every business and every corner of the globe has been affected. Hundreds of millions of jobs have been lost . . . Many small and medium-sized enterprises—the engine of the global economy—may not survive.

After painting a depressing picture of the future and explaining how difficult it will be for the world economy to return to “normal,” Guterres’s (2020) address makes a hardly perceptible turn when he says “let’s not forgot the pre-Covid-19 world was far from normal.” It seems then that, rather than shattering the world of work as we know it, the COVID-19 pandemic simply exposes in higher resolution the “tremendous shortcomings, fragilities and fault lines” that have been eroding society and economy from the bottom-up for decades. The pre-COVID-19 world, in which we thought we lived a normal life, is not as normal as we think (see Guterres, 2020).

To save the economy under siege is to “return to normal” as soon as possible, so cry the bureaucrats and journalists alike. But what is “normal” in this case? What does “being normal” mean exactly? Is the world, old or new, ever normal? There are norms regulating life, but has there ever been a “normal life” as such? The so-called normal life, a life before COVID-19, to which we pray to return, is in truth one of recollection, a romantic one at that, as the UN policy brief readily admits. Just as a viral infection may display more than one symptom on the part of its victims, embody more than one single illness, and create more than one single public health challenge, life, as it is actually lived, is hardly reducible to one normal life. In fact, the so-called normal life is the one that brought us the pandemic in the first place. To live is to live normally; to return to normal is what living is all about. The so-called new normal is both new and not so new, which is to say, it is neither really new nor really normal. Perhaps the world has never been and will never be normal, whatever our idea of “being normal” means. If a pandemic can turn the world upside down, it is because life has been *turning and turning again.* And anything that *re*turns cannot be entirely new.

### Pandemics, quo vadis?

Humans have been haunted by viruses since time immemorial. From the prehistoric pandemics in northeastern China 5,000 years ago, uncovered at sites now called Hamin Mangha and Miaozigou, to the Justinian plague (541–549 AD) that may have helped to bring down feudalism, or the small pox outbreak that finally toppled the Aztec empire before Hernán Cortés returned to the region in the spring of 1521, viral infections have tormented the lands and their people over millennia. Traveling with host animals and humans, viruses had gone global long before globalization became a fact.

There are 10 known pandemics in the last 250 years, all displaying the same pattern of spiking in seasonal waves after the initial attacks. COVID-19, and some of its coronal cousins, will undoubtedly expand the list. To those who are living through its assault, the impacts brought about by COVID-19 are more or less clear and more or less measurable. But what is the meaning of COVID-19 when the current pandemic is over? Will it be remembered? If so, in what way and to what extent? If the history of pandemics has taught us anything, it is that history tends to repeat itself, that viral outbreaks are an ineliminable part of the natural history, in which humans are a part and in which no “zone of being” is free from viral infection. Recall the Spanish Flu of 1918, the worst pandemic during the last two centuries. It is estimated to have wiped out 50 million people worldwide, meanwhile infecting 500 million, a third of the world’s population at the time. However, despite its short distance of mere one hundred years from us, few people today know much about it, and still fewer are able to understand or feel the impact it had at that time. Its centenary a short time ago passed noiselessly, certainly not for lack of stories or records. Like the many plagues before it, the Spanish Flu, it seems, never quite made itself into what Reinhart Koselleck (2018) calls the “the space of experience” (p. 34). Failing to make its way into collective memory, it is also helpless in figuring into our “horizon of expectation” (Koselleck, 2018, p. 14). If the Spanish Flu faded largely from memory, all the woes caused by COVID-19 are, likewise, likely to dissipate in time, regardless of how we feel and say about it now. There was a pre-coronavirus world, and there will be a post-coronavirus world, but viruses, known or novel, will outlast our worlds.

Viruses are everywhere and forever. So, plagues will never disappear for good (Camus, 1991, p. 307). But what then does it mean, the pandemic? It is life, that is all. A troubled memory, fading, under the vast indifference of the sky. Until the gate of Oran closes again.

## Conclusion (Xuefeng Feng and Briankle G. Chang)

Assembled in this forum, the five short essays provide some modest reflections on the coronavirus pandemic and its still unfolding consequences. Committed to a variety of disciplinary perspectives and interests, the authors did not set out by pursuing any preset direction or common agenda supposedly carried out collectively in our intellectual labor. Rather, what unifies the diverse inquiries in these essays is the shared awareness about the confusion in the public discourse that constantly fails to distinguish a coronavirus called COVID-19 from the COVID-19 pandemic, or as Briankle reminds us in his essay, from “a series of localized epidemics.” This alertness constitutes a common ground in addressing specific issues or phenomena in these essays.

This forum is anything but comprehensive. If it can contribute to the discussion of the crisis, it is most likely because all the essays refuse to bind the pandemic exclusively with the coronavirus and to position the virus and humanity in rigid opposition to each other. In her multispecies ethnography, Anna Tsing tells a marvelous story about matsutake mushrooms. “When Hiroshima was destroyed by an atomic bomb in 1945,” says she, “it is said, the first living thing to emerge from the blasted landscape was a matsutake mushroom” (Tsing, 2015, p. 3). When human history temporarily comes to a halt in disasters, matsutake, and also viruses in our case, may well survive and continue to thrive with their own stories. Histories are being made every day by humans and non-humans alike; however, the future for those histories to converge has still yet to come. As demonstrated in the essays gathered here, while governments and the public are desperate to frame the virus in their own social and political narratives, the virus also works hard to inscribe its historicity on the earth and humans too.

If a message must be dispatched out to all at this juncture, it is that for a future of collaborative survival, the stake of living together has nothing to do with harmony and conquest, but is derived from “disturbance-based ecologies” (Tsing, 2015, p. 5), that is, plagues. Plagues are life, that is all.

## Notes

1. In a trilogy of contemporary essays, Baudrillard (1995) argued that the war in the desert was a new phenomenon, because it was defined and shaped by its discursive aspect as a form of television programming, regardless of what happened on the ground.

2. That earlier announcement called “demonetisation” led to a vast contraction of commerce and an immiseration of a majority of the population which has still not recovered, and is now widely considered an unnecessary exercise of personal whimsy. For an analysis of the economic consequences, see Ghosh et al. (2017).

3. The decline of Indian agriculture is not adequately discussed in the celebration of urban growth. Its effects are however inescapable and directly lead to the growth of informal settlements in cities (Balakrishnan, 2020; Jaffrelot & Thakker, 2020).

4. Mediatization, or the analysis of events with their mediated construction as the starting point, is a phenomenon that has grown in importance across cultural contexts, as media theorists attempt to understand the increasing influence of media forms on culture, especially with the virtualization of human interaction and the redefinition of community through the use of social media and mobile communication. For a fuller discussion, see Couldry and Hepp (2013).

5. Re-examining her arguments in *Citizenship and Its Discontents: An Indian History* (2013), Jayal (2019) traces the current shift in the discourse of Indian citizenship from an egalitarian rights-based model to a new regime predicated on religious and cultural identity, given shape through the concomitant technocratic frames of transactional welfare and biometric identity.

6. Four short essays provide further context and narrate the response: Shankar et al. (2020).

7. Partha Chatterjee’s (2004, 2011) insightful categorization of Indian society separates the distinct ontological domain of a small formal “civil society” that includes rights-bearing *citizens*, from the vast undifferentiated mass of the *population* that constitutes “political society” and which forms the actual locus of democratic practice.

8. The current Indian government has dramatically increased the acquisition and use of big data in governance, including a reliance on biometric identification for access to welfare programs and the use of mobile phones for access to services: Part of the government’s COVID-19 response was in the form of a mandatory mobile application that purported to use location tracking to show active virus infections in the user’s vicinity. An analysis of its invasive nature can be found in a working paper by Deb (2020).

9. Societies always declared war on problems they cannot solve: war on drugs, war on poverty, and the like. It is no surprise to see Donald Trump refers to COVID-19 as an “invisible enemy” and calls himself a war-time president in his speeches on the COVID-19 pandemic. Similar examples abound across the board throughout history. It is widely recognized by epidemiologists today that the model developed by John Snow based on the cholera outbreaks between 1831 and 1853 in England is too linear to be of any use in contemporary pandemics. Like the global climate instability or financial market volatility, pandemics are nonlinear phenomena, displaying a high degree of uncertainty that defies simple causal explanation. On this, see Kosner (2020).

10. It is therefore not surprising that we observe Donald Trump repeatedly refers to COVID-19 as Wuhan virus or Kung Flu in reaction to his rise and fall in poll and public opinion as he tries to find scapegoat, in this case, China, for his sorry failure in handling the crisis. A virus is always more than a virus when it enters the body politic.
